# Medical contrast media as possible tools for SAXS contrast variation

**DOI:** 10.1107/S2052252519005943

**Published:** 2019-05-29

**Authors:** Frank Gabel, Sylvain Engilberge, Javier Pérez, Eric Girard

**Affiliations:** a IBS, CEA, CNRS, UGA, 71 avenue des Martyrs, 38000 Grenoble, France; b Synchrotron SOLEIL, Saint-Aubin BP 48, 91192 Gif-sur-Yvette, France

**Keywords:** SAXS, medical contrast media, contrast variation, macromolecular complexes, electron densities, soft-matter systems, structural information

## Abstract

Small-angle X-ray scattering (SAXS) is increasingly used to extract structural information from a multitude of soft-matter and biological systems in aqueous solution, including polymers, detergents, lipids, colloids, proteins and RNA/DNA. When SAXS data are recorded at multiple contrasts, *i.e.* at different electron densities of the solvent, the internal electron-density profile of solubilized molecular systems can be probed. However, contrast-variation SAXS has been limited by the range of electron densities available by conventional agents such as sugars, glycerol and salt, and by the fact that many soft-matter and biological systems are modified in their presence. Here we present a pioneering SAXS contrast-variation study on DDM (*n*-do­decyl-β-d-malto­pyran­oside) micelles by using two highly electron-rich contrast agents from biomedical imaging which belong to the families of gadolinium-based and iodinated molecules. The two agents, Gd-HPDO3A and iohexol, were allowed to attain modifications of the solvent electron density that are 50 to 100% higher than those obtained for sucrose, and are located between the electron densities of proteins and RNA/DNA. In the case of Gd-HPDO3A, an analysis of the internal micellar structure was possible and compared with results obtained with sucrose. In conclusion, medical contrast agents represent a promising class of molecules for SAXS contrast-variation experiments with potential appli­cations for numerous soft-matter and biological systems, including membrane proteins and protein–RNA/DNA complexes.

## Abstract 2

Medical contrast media are presented as possible tools to probe the internal structure of soft-matter and biological systems by small-angle X-ray scattering solvent contrast variation.

## 1. Introduction   

Small-angle X-ray scattering (SAXS) has been used for several decades to extract structural information from a multitude of soft-matter and biological systems in aqueous solution, including polymers, detergents, lipids, colloids, proteins and RNA/DNA (Glatter, 2018 ▸; Svergun *et al.*, 2013 ▸; Putnam *et al.*, 2007 ▸; Lipfert & Doniach, 2007 ▸; Lindner & Zemb, 2002 ▸). It is sensitive to the electron-density difference between the solubilized particles (ρ) and the solvent (ρ_sol_) in the nano to micrometre range.

The internal electron-density distribution of composite solubilized particles (*i.e.* particles composed of segregated zones of different electron density) can by probed efficiently with SAXS experiments by varying ρ_sol_. Historically, several small electron-rich molecules have been used to modify the solvent electron density, including sucrose (Garcia-Diez *et al.*, 2016 ▸; Bolze *et al.*, 2003 ▸; Kiselev *et al.*, 2001 ▸; Ballauff, 2001 ▸; Dingenouts & Ballauff, 1993 ▸), glycerol (Hickl *et al.*, 1996 ▸; Bolze *et al.*, 1996 ▸) and salt (Naruse *et al.*, 2009 ▸; Fernandez *et al.*, 2008 ▸). The value of ρ_sol_ can usually be varied between 0.335 e Å^−3^ (pure water) and a maximum of about 0.41 e Å^−3^, which has been reached for ‘conventional’ contrast agents, *e.g.* sucrose at 1.8–2.0 *M* [50–55%(*w*/*w*)] (Jeffries *et al.*, 2016 ▸; Kiselev *et al.*, 2003 ▸, 2001 ▸; Dingenouts & Ballauff, 1993 ▸; Kirste & Stuhrmann, 1967 ▸) and 100% glycerol (Wolf *et al.*, 1989 ▸; Kirste & Stuhrmann, 1967 ▸). High molar NaCl solutions provide electron densities up to 0.38 e Å^−3^ but in practice are problematic since they may perturb the structural integrity of many systems (Chen *et al.*, 2017 ▸; Naruse *et al.*, 2009 ▸; Fernandez *et al.*, 2008 ▸).

**Figure 1:**
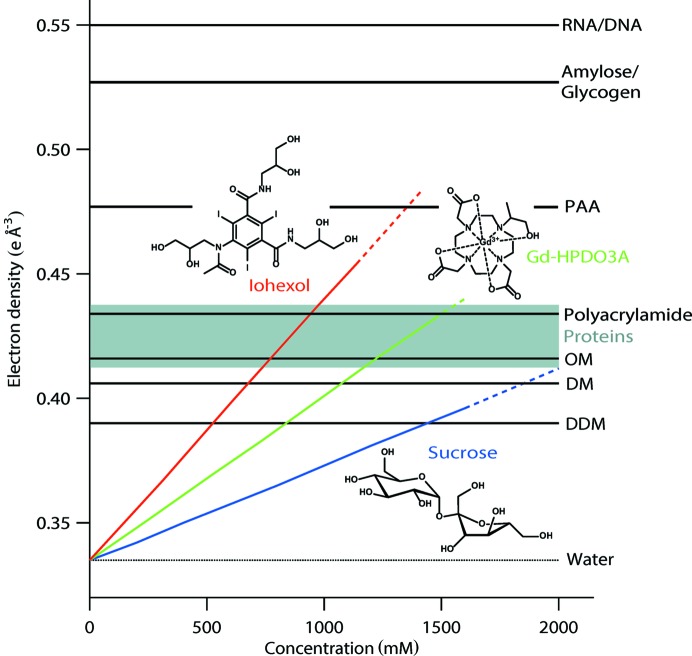
Electron densities of iohexol, Gd-HPDO3A and sucrose solutions, and their comparison with various soft-matter systems and biological molecules (PAA, polyacrylic acid; OM, *n*-octyl-β-d-malto­pyran­oside; DM, decyl maltoside) (Durchschlag & Zipper, 1994 ▸). Continuous lines denote solute concentrations covered in the present study (automatic injection in flow mode on a SAXS beamline), broken lines denote solubility limits reported in the literature (Rickwood *et al.*, 1982 ▸) and determined from our own solubilization assays for batch samples.

As a consequence, the electron density of many important molecular systems (Fig. 1 ▸) cannot be attained with conventional contrast agents, so there is interest in developing new compounds for improved SAXS contrast variation. Practical requirements of such compounds include elevated electron density, high solubility and chemical inertness towards the molecular systems to be studied.

Here, we study two highly electron-rich medical contrast media, iohexol and Gd-HPDO3A, in SAXS experiments on DDM (*n*-do­decyl-β-d-malto­pyran­oside) solutions and compare their performance with sucrose solutions. Iohexol (C_19_H_26_I_3_N_3_O_9_), commercialized under the trade names Omnipaque or Histodenz (among others), is a non-ionic tri-iodinated molecule used as an X-ray imaging contrast medium. Gd-HPDO3A (C_17_H_29_GdN_4_O_7_) or Gadoteridol (trade name ProHance) is a neutral paramagnetic contrast agent for magnetic resonance imaging that contains a single gadolinium atom.

Aqueous solutions of both media readily attain electron densities between 0.43 and 0.46 e Å^−3^ at moderate concentrations, equivalent to a 50–100% increase in solvent electron density compared with sucrose (Fig. 1 ▸). The elevated electron densities reached allow a detailed study of the internal structure of DDM micelles in the case of Gd-HPDO3A, in agreement with the results obtained in sucrose for lower contrast values. In the case of iohexol, specific interactions with DDM micelles were observed at higher concentrations.

Importantly, the electron densities reached here cover an unprecedented range of soft-matter systems and biological molecules, including proteins, and approach those of nucleotides (RNA/DNA). Medical contrast media are therefore a promising class of molecules for SAXS contrast variation with a high potential to expand the range and applications of this technique to a multitude of soft-matter and biomolecular systems.

While DDM micelles were used here as an appropriate model system to characterize and calibrate SAXS contrast properties of two medical contrast media, further studies are required for more routine applications. In particular, a broader range of these media should be probed in multiple soft-matter and biological systems in order to characterize and, if possible, improve issues observed in the present study, notably the specific interaction with solubilized particles and the strong X-ray absorbance.

## 2. Results   

### 2.1. Medical contrast media attain electron densities inaccessible to conventional SAXS contrast agents   

**Figure 2:**
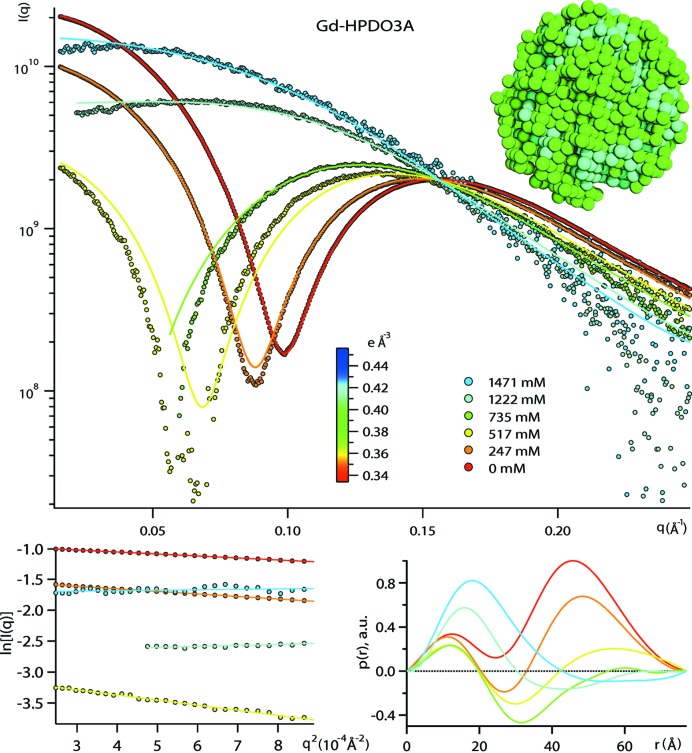
Experimental DDM scattering curves and *MONSA* fits at various Gd-HPDO3A concentrations (top) and the resulting *ab initio* DDM micelle model (green beads, head groups; cyan beads, tail groups). Guinier plots (bottom left) and *p*(*r*) functions (bottom right).

In order to evaluate the potential of medical contrast media as contrast agents for SAXS experiments, we compared the following properties of DDM micelles in iohexol, Gd-HPDO3A and sucrose solutions: solvent electron densities (Fig. 1 ▸ and the Supporting information), SAXS curves and contrast match points (Fig. 2 ▸, and Figs. S1 and S4 in the Supporting information), and low resolution *ab initio* shapes (Figs. 2 ▸ and S5).

Both medical contrast media readily outperformed sucrose as contrast agents: Gd-HPDO3A (1471 m*M*) and iohexol (1138 m*M*) solutions yielded maximum solvent electron densities of 0.425 and 0.455, respectively, compared with 0.395 e Å^−3^ for sucrose (1600 m*M*) (Fig. 1 ▸). In other words, Gd-HPDO3A and iohexol increase the electron density of pure water (0.335 e Å^−3^) 1.6 and 2.8 times more efficiently (per mol l^−1^), respectively, than sucrose: 0.0612 and 0.1055 versus 0.0375 e Å^−3^ *M* ^−1^, respectively.

The contrast match points [*I*(0) = 0] yielded identical electron densities for DDM micelles (0.387 ± 0.005) in sucrose, Gd-HPDO3A and iohexol (Fig. S4). Importantly, the concentrations required to match DDM were significantly lower for the medical contrast media (853 and 498 m*M* for Gd-HPDO3A and iohexol, respectively) than for sucrose (1392 m*M*), illustrating the enhanced capacity of these molecules to modify contrast when compared with conventional molecules. Expressed as weight/weight (*w*/*w*) fractions (*cf*. Supporting information), the values of the DDM contrast match points were 40.5, 39.5 and 34.0% in sucrose, Gd-HPDO3A and iohexol, respectively.

### 2.2. Chemically inert contrast media allow a detailed analysis of DDM micelle structures   

Basic parameters [radii of gyration *R*_G_, maximum dimensions *D*_max_ and pair distance distribution functions *p*(*r*)] of DDM micelles were extracted for all SAXS curves in various concentrations of sucrose, Gd-HPDO3A and iohexol (Figs. 2 ▸ and S1, and Table S4). The (apparent) aggregation numbers *N*_agg_ were determined from the *I*(0) intensities [equation (S5)] and found to lie between 129 and 156 (Table S5).

In the case of sucrose and Gd-HPDO3A, the micelles were modelled by *MONSA* (Svergun, 1999 ▸) multi-phase *ab initio* shape determination (Figs. 2 ▸ and S5). In the case of iohexol, the approach was attempted but failed (Fig. S5). As this modelling approach requires that the structure remains the same for all contrast conditions, failure could be because of a progressive shape deformation of the micelles, as suggested by the *p*(*r*) functions that did not yield a stable *D*_max_ at higher iohexol concentrations (Fig. S1 and Table S4). An alternative explanation could be a fusion of a single (or a few) iohexol molecule into the micelles, below the sensitivity level of *I*(0). *MONSA* *ab initio* modelling was applied to the entire contrast series in sucrose and Gd-HPDO3A, and yielded non-spherical particles, with a clear core-shell separation of the two phases (Figs. 2 ▸ and S5). While the cores (half-axes *a* and *b*) displayed slightly irregular shapes (*i.e.* protuberances) in the case of sucrose, they were more regular in the case of Gd-HPDO3A. Overall, the shapes of micelle models from sucrose and Gd-HPDO3A corresponded to oblate, symmetric core-shell ellipsoids with half-axes *b* + *t* = 37.0 ± 2.0 Å, *a* + *t* = 26.0 ± 2.0 Å and a shell thickness of *t* = 6.0 ± 2.0 Å, for both sucrose and Gd-HPDO3A. Not surprisingly, the use of a single contrast condition did not generate a reasonable core-shell phase separation (Fig. S6).

## 3. Discussion   

The ensemble of data presented here demonstrate that certain medical contrast media are excellent candidates for SAXS contrast-variation experiments, reaching unprecedented solvent electron densities inaccessible to ‘conventional’ contrast agents such as sucrose (Fig. 1 ▸). Indeed, Gd-HPDO3A solutions readily attain electron densities of 0.43 e Å^−3^, thus covering a wide range of soft-matter systems, including detergents (DDM, DM and OM) and biomacromolecules (proteins). Iohexol solutions even reach electron densities up to 0.46 e Å^−3^, located between those of proteins and nucleic acids (RNA, DNA). However, while iohexol does not induce a more significant change in the aggregation state of DDM molecules when compared with sucrose and Gd-HPDO3A (Table S5), it appears to perturb the overall shape of the micelles at higher concentrations (Figs. S1 and S5) and can therefore not be considered as an ‘inert’ contrast agent for this specific system.

The experimental contrast match points of DDM in sucrose, Gd-HPDO3A and iohexol (Fig. S4) indicate an average electron density of 0.387 ± 0.005 e Å^−3^ for the micelles. This value is close to the lower limit of electron densities (0.392–0.405 e Å^−3^) reported for DDM in aqueous solution or calculated from the molecular volume (Lipfert *et al.*, 2007 ▸; le Maire *et al.*, 2000 ▸). However, since SAXS integrates over the entire volume where electron densities of a dissolved particle differ from those of the bulk solvent, the average particle electron density can potentially also include specific hydration effects (Kim *et al.*, 2016 ▸; Svergun *et al.*, 1998 ▸). Indeed, sedimentation equilibrium experiments performed by analytical ultracentrifugation (AUC) have shown that hydrated DDM micelles have an apparent lower density than non-hydrated micelles in concentrated sucrose and iohexol solutions (Lustig *et al.*, 2000 ▸). However, the apparent electron densities reported in the AUC study are significantly lower (∼0.37 e Å^−3^) than those in the present work and a conclusion on DDM hydration properties can therefore not be drawn. A detailed analysis of potential hydration effects may be possible with recently developed molecular dynamics approaches which include explicit solvent (Ivanović *et al.*, 2018 ▸).

*Ab initio* modelling with *MONSA* (Figs. 2 ▸, S5 and S6) provided oblate ellipsoidal shapes with well separated core and shell phases in the case of sucrose and Gd-HPDO3A. The overall dimensions of the ellipsoidal axes and the shell thickness are in good agreement with the *D*_max_ from the *p*(*r*) functions (Table S4). The DDM micelle cores in Gd-HPDO3A do not display the protuberances that occur occasionally in models from the sucrose data (Fig. S5). As expected, a single SAXS contrast is insufficient to carry out a *MONSA* modelling successfully (Fig. S6), underlining the interest of measuring a contrast series. It is important to stress that the *MONSA* models should be considered as an average micelle model, integrating over potential size and shape polydispersity.

In the SAXS contrast-variation study presented here, medical contrast media were used on DDM micelles as a model system. The iohexol data (Figs. 1 ▸ and S4), in particular, illustrate that this class of molecules has huge potential for biological macromolecules in terms of elevated electron density. Indeed, recent SAXS contrast-variation studies on proteins (Jeffries *et al.*, 2016 ▸; Schneidman-Duhovny *et al.*, 2013 ▸; Grishaev *et al.*, 2012 ▸) or protein–DNA complexes (Chen *et al.*, 2017 ▸, 2014 ▸; Tokuda *et al.*, 2016 ▸; Pollock *et al.*, 2016 ▸; Inoko *et al.*, 1992 ▸) were carried out with sucrose and salt solutions and were therefore limited to solvent electron densities which just manage to match those of proteins.

The contrast media studied here are able to go significantly beyond that density, well into the range between proteins and RNA/DNA (0.41–0.55 e Å^−3^). They therefore open new and exciting perspectives for SAXS contrast-variation experiments on biomacromolecular systems such as membrane proteins or protein–RNA/DNA complexes that were so far out of reach for X-rays and exclusively within the domain of small-angle neutron scattering (SANS) (Mahieu & Gabel, 2018 ▸, Gabel, 2015 ▸, Chaudhuri, 2015 ▸). However, SANS will probably remain superior to SAXS in its capacity to globally label (by deuteration), and therefore distinguish chemically similar molecules (*e.g.* different proteins in a reconstituted complex). Finally, the strong absorbance of X-rays by heavy atoms contained in medical contrast media provides an efficient and handy tool to calibrate and validate concentrations *in situ* during SAXS experiments (Fig. S3).

As illustrated by the interaction of iohexol and DDM micelles in the present work, medical contrast media may affect the structural integrity of the solubilized particles being studied. The inertness of SAXS contrast agents therefore needs to be studied on a case-by-case basis, in analogy to SANS experiments, where the effect of D_2_O on the oligomeric state of biomacromolecules and their complexes is probed routinely by monitoring *I*(0) as a function of contrast (Trewhella *et al.*, 2017 ▸; Jacrot, 1976 ▸).

Efforts to improve the efficiency of new molecules as SAXS contrast agents should therefore focus on designing systems with (1) elevated electron density, (2) high solubility and low viscosity, and (3) chemical inertness towards the soft-matter and biological systems to be studied. Additional ways to improve the quality of SAXS contrast-variation data include the use of thinner samples (with more frames being binned); photon flux increase with the implementation of large bandwidth monochromators, associated with co-flow devices for capillary fouling reduction (Kirby *et al.*, 2016 ▸); and the use of high-efficiency noiseless counting detectors.

## 4. Materials and methods summary   

Iohexol (catalogue number D2158) and sucrose (catalogue number S7903) were purchased from Sigma–Aldrich (St Louis, Missouri, USA). DDM (*n*-do­decyl-β-d-malto­pyran­oside) was purchased from Anatrace (Maumee, Ohio, USA) (catalogue number D310). Gd-HPDO3A was kindly provided by Bracco Imaging SpA (Milan, Italy). All SAXS contrast agent stock solutions were prepared in Milli-Q water and sequentially diluted to reach the final concentration. For each measurement DDM was weighed on a high-precision balance and solubilized by the required contrast-agent solution. All SAXS stock solutions were prepared by adding Milli-Q water to appropriate amounts of contrast agent. In order to reach the final concentration, DDM was weighed on a high-precision balance and added in an appropriate amount to reach a concentration of 20 mg ml^−1^. Full details of SAXS sample preparation are provided in the Supporting information.

All SAXS experiments were carried out on the SWING beamline (https://www.synchrotron-soleil.fr/en/beamlines/swing) at the synchrotron SOLEIL (Saint-Aubin, France) in flow mode, using an X-ray energy of 12.00 keV and a sample to detector distance of 1.790 m. For each sample a volume of 40 µl was circulated at 75 µl min^−1^ through a thermalized Quartz capillary of 1.5 mm diameter and 10 µm wall thickness, inserted within a vacuum chamber (David & Pérez, 2009 ▸). Individual 1 s time frames were collected at 15°C. The 2D scattering patterns were reduced into 1D intensities and selected for averaging using the *Foxtrot* software (https://www.synchrotron-soleil.fr/en/beamlines/swing#paragraphes_menu_left-block-6). Buffer intensities were subtracted from sample intensities using the program *PRIMUS* (Franke *et al.*, 2017 ▸) after careful calibration against the measured transmissions. Basic parameters [radii of gyration *R*_G_, pair distance distribution functions *p*(*r*), and maximum dimensions *D*_max_] were determined by *PRIMUS*. *Ab initio* shape analysis was carried out by the program *MONSA* (Svergun, 1999 ▸). Full details of SAXS data reduction and analysis are provided in the Supporting information.

## 5. Related literature   

The following references are cited in the Supporting information for this article: Appolaire *et al.* (2014 ▸), Breyton *et al.* (2013 ▸), Stuhrmann (1974) ▸ and Weast (1989) ▸.

## Supplementary Material

Supporting information - tables and figures. DOI: 10.1107/S2052252519005943/tj5021sup1.pdf

## References

[bb1] Appolaire, A., Girard, E., Colombo, M., Durá, M. A., Moulin, M., Härtlein, M., Franzetti, B. & Gabel, F. (2014). *Acta Cryst.* D**70**, 2983–2993. 10.1107/S1399004714018446 PMC4220976 25372688

[bb2] Ballauff, M. (2001). *Curr. Opin. Colloid Interface Sci.* **6**, 132–139.

[bb3] Bolze, J., Ballauff, M., Kijlstra, J. & Rudhardt, D. (2003). *Macromol. Mater. Eng.* **288**, 495–502.

[bb4] Bolze, J., Hörner, K. D. & Ballauff, M. (1996). *Langmuir*, **12**, 2906–2912.

[bb5] Breyton, C., Gabel, F., Lethier, M., Flayhan, A., Durand, G., Jault, J.-M., Juillan-Binard, C., Imbert, L., Moulin, M., Ravaud, S., Härtlein, M. & Ebel, C. (2013). *Eur. Phys. J. E*, **36**, 71. 10.1140/epje/i2013-13071-6 23852580

[bb6] Chaudhuri, B. N. (2015). *Protein Sci.* **24**, 267–276. 10.1002/pro.2624 PMC4353354 25516491

[bb7] Chen, Y., Tokuda, J. M., Topping, T., Meisburger, S. P., Pabit, S. A., Gloss, L. M. & Pollack, L. (2017). *Proc. Natl Acad. Sci. USA*, **114**, 334–339. 10.1073/pnas.1611118114 PMC5240728 28028239

[bb8] Chen, Y., Tokuda, J. M., Topping, T., Sutton, J. L., Meisburger, S. P., Pabit, S. A., Gloss, L. M. & Pollack, L. (2014). *Nucleic Acids Res.* **42**, 8767–8776. 10.1093/nar/gku562 PMC4117781 24990379

[bb9] David, G. & Pérez, J. (2009). *J. Appl. Cryst.* **42**, 892–900.

[bb10] Dingenouts, N. & Ballauff, M. (1993). *Acta Polym.* **44**, 178–183.

[bb11] Durchschlag, H. & Zipper, P. (1994). *Prog. Colloid. Polym. Sci.* **94**, 20–39.

[bb12] Fernandez, R. M., Riske, K. A., Amaral, L. Q., Itri, R. & Lamy, M. T. (2008). *Biochim. Biophys. Acta*, **1778**, 907–916. 10.1016/j.bbamem.2007.12.005 18178145

[bb13] Franke, D., Petoukhov, M. V., Konarev, P. V., Panjkovich, A., Tuukkanen, A., Mertens, H. D. T., Kikhney, A. G., Hajizadeh, N. R., Franklin, J. M., Jeffries, C. M. & Svergun, D. I. (2017). *J. Appl. Cryst.* **50**, 1212–1225. 10.1107/S1600576717007786 PMC5541357 28808438

[bb14] Gabel, F. (2015). *Methods Enzymol.* **558**, 391–415. 10.1016/bs.mie.2015.02.003 26068748

[bb15] Garcia-Diez, R., Sikora, A., Gollwitzer, C., Minelli, C. & Krumrey, M. (2016). *Eur. Polym. J.* **81**, 641–649.

[bb16] Glatter, O. (2018). *Scattering Methods and their Application in Colloid and Interface Science.* Amsterdam: Elsevier.

[bb17] Grishaev, A., Anthis, N. J. & Clore, G. M. (2012). *J. Am. Chem. Soc.* **134**, 14686–14689. 10.1021/ja306359z PMC3442789 22908850

[bb18] Hickl, P., Ballauff, M. & Jada, A. (1996). *Macromolecules*, **29**, 4006–4014.

[bb21] Inoko, Y., Yamamoto, M., Fujiwara, S. & Ueki, T. (1992). *J. Biochem.* **111**, 310–316. 10.1093/oxfordjournals.jbchem.a123755 1587792

[bb22] Ivanović, M. T., Bruetzel, L. K., Lipfert, J. & Hub, J. S. (2018). *Angew. Chem. Int. Ed.*, **57**, 5635–5639. 10.1002/anie.201713303 29532982

[bb23] Jacrot, B. (1976). *Rep. Prog. Phys.* **39**, 911–953.

[bb24] Jeffries, C. M., Graewert, M. A., Blanchet, C. E., Langley, D. B., Whitten, A. E. & Svergun, D. I. (2016). *Nat. Protoc.* **11**, 2122–2153. 10.1038/nprot.2016.113 PMC5402874 27711050

[bb25] Kim, H. S., Martel, A., Girard, E., Moulin, M., Härtlein, M., Madern, D., Blackledge, M., Franzetti, B. & Gabel, F. (2016). *Biophys. J.* **110**, 2185–2194. 10.1016/j.bpj.2016.04.013 PMC4880798 27224484

[bb26] Kirby, N., Cowieson, N., Hawley, A. M., Mudie, S. T., McGillivray, D. J., Kusel, M., Samardzic-Boban, V. & Ryan, T. M. (2016). *Acta Cryst.* D**72**, 1254–1266. 10.1107/S2059798316017174 PMC5137223 27917826

[bb27] Kirste, R. G. & Stuhrmann, H. B. (1967). *Z. Physik. Chemie.* **56**, 338.

[bb28] Kiselev, A. M., Lesieur, P., Kisselev, A. M., Lombardo, D., Killany, M. & Lesieur, S. (2001). *J. Alloys Compd*, **328**, 71–76.

[bb29] Kiselev, M. A., Wartewig, S., Janich, M., Lesieur, P., Kiselev, A. M., Ollivon, M. & Neubert, R. (2003). *Chem. Phys. Lipids*, **123**, 31–44. 10.1016/s0009-3084(02)00140-8 12637163

[bb30] Lindner, P. & Zemb, T. (2002). *Neutrons, X-rays and Light: Scattering Methods Applied to Soft Condensed Matter.* Amsterdam: Elsevier.

[bb31] Lipfert, J., Columbus, L., Chu, V. B., Lesley, S. A. & Doniach, S. (2007). *J. Phys. Chem. B*, **111**, 12427–12438. 10.1021/jp073016l 17924686

[bb32] Lipfert, J. & Doniach, S. (2007). *Annu. Rev. Biophys. Biomol. Struct.* **36**, 307–327. 10.1146/annurev.biophys.36.040306.132655 17284163

[bb33] Lustig, A., Engel, A., Tsiotis, G., Landau, E. M. & Baschong, W. (2000). *Biochim. Biophys. Acta*, **1464**, 199–206. 10.1016/s0005-2736(99)00254-0 10727607

[bb34] Mahieu, E. & Gabel, F. (2018). *Acta Cryst.* D**74**, 715–726. 10.1107/S2059798318005016 30082507

[bb35] Maire, M. le, Champeil, P. & Møller, J. V. (2000). *Biochim. Biophys. Acta*, **1508**, 86–111. 10.1016/s0304-4157(00)00010-1 11090820

[bb36] Naruse, K., Eguchi, K., Akiba, I., Sakurai, K., Masunaga, H., Ogawa, H. & Fossey, J. S. (2009). *J. Phys. Chem. B*, **113**, 10222–10229. 10.1021/jp9019415 19572674

[bb37] Pollock, N. L., Satriano, L., Zegarra-Moran, O., Ford, R. C. & Moran, O. (2016). *J. Struct. Biol.* **194**, 102–111. 10.1016/j.jsb.2016.02.004 26850167

[bb38] Putnam, C. D., Hammel, M., Hura, G. L. & Tainer, J. A. (2007). *Q. Rev. Biophys.* **40**, 191–285. 10.1017/S0033583507004635 18078545

[bb39] Rickwood, D., Ford, T. & Graham, J. (1982). *Anal. Biochem.* **123**, 23–31. 10.1016/0003-2697(82)90618-2 7114474

[bb40] Schneidman-Duhovny, D., Hammel, M., Tainer, J. A. & Sali, A. (2013). *Biophys. J.* **105**, 962–974. 10.1016/j.bpj.2013.07.020 PMC3752106 23972848

[bb41] Stuhrmann, H. B. (1974). *J. Appl. Cryst.* **7**, 173–178.

[bb42] Svergun, D. I. (1999). *Biophys. J.* **76**, 2879–2886. 10.1016/S0006-3495(99)77443-6 PMC1300260 10354416

[bb43] Svergun, D. I., Koch, M. H. J., Timmins, P. A. & May, R. P. (2013). *Small Angle X-ray and Neutron Scattering from Solutions of Biological Macromolecules.*, Oxford University Press.

[bb44] Svergun, D. I., Richard, S., Koch, M. H. J., Sayers, Z., Kuprin, S. & Zaccai, G. (1998). *Proc. Natl Acad. Sci. USA*, **95**, 2267–2272. 10.1073/pnas.95.5.2267 PMC19315 9482874

[bb45] Tokuda, J. M., Pabit, S. A. & Pollack, L. (2016). *Biophys. Rev.* **8**, 139–149. 10.1007/s12551-016-0196-8 PMC4991782 27551324

[bb46] Trewhella, J., Duff, A. P., Durand, D., Gabel, F., Guss, J. M., Hendrickson, W. A., Hura, G. L., Jacques, D. A., Kirby, N. M., Kwan, A. H., Pérez, J., Pollack, L., Ryan, T. M., Sali, A., Schneidman-Duhovny, D., Schwede, T., Svergun, D. I., Sugiyama, M., Tainer, J. A., Vachette, P., Westbrook, J. & Whitten, A. E. (2017). *Acta Cryst.* D**73**, 710–728. 10.1107/S2059798317011597 PMC5586245 28876235

[bb47] Weast, R. C. (1989). Editor. *Handbook of Chemistry and Physics.* Boca Raton, Florida: CRC Press.

[bb48] Wolf, A. V., Brown, M. G. & Prentiss, P. G. (1989). *Handbook of Chemistry and Physics.* 70th ed. Boca Raton, Florida: CRC Press.

