# Supplementary material for: Medical contrast media as possible tools for SAXS contrast variation
Source: IUCrJ. 2019 May 29;6(Pt 4):521–5. doi: 10.1107/S2052252519005943 (PMC6608644; doi:10.1107/S2052252519005943)
Supplement: Supplementary file 1 [file m-06-00521-sup1.pdf]

IUCrJ

Volume 6 (2019)

Supporting information for article:

Medical contrast media as possible tools for SAXS contrast variation

Frank Gabel, Sylvain Engilberge, Javier Pérez and Eric Girard

Sample preparation

Iohexol (catalogue number D2158) and sucrose (catalogue number S7903) were purchased from Sigma-Aldrich. DDM (n-Dodecyl-β-D-Maltopyranoside) was purchased from Anatrace (catalogue number D310). Gd-HPDO3A was kindly provided by Bracco Imaging S.p.A., Milan, Italy. All SAXS contrast agent stock solutions were prepared by weighing a given amount of contrast agent on a high-precision balance and adding Milli-Q water by pipetting. The final volume of the mixture was measured by pipetting. The stock solutions were sequentially mixed in appropriate volume ratios with Milli-Q water to reach the desired concentration for a final sample volume of 50 µL. An example of a detailed protocol and amounts of material mixed is given in Table S1 for 1 M reference solutions that were used to calculate and calibrate electron densities, including those of other samples (see below). For samples containing DDM, a high precision balance was used to weigh 1 mg of the detergent powder in an Eppendorf tube. Subsequently, 50 µL of the contrast solution at the respective concentration was added and gently mixed with the DDM powder by pipetting.

Calculation of solvent electron densities

The solvent electron densities (ρ_{sol}) were calculated from the amounts of sucrose ($C_{12}H_{22}O_{11}$; 342.3 Da; 182 e^-), Gd-HPDO3A ($C_{17}H_{29}GdN_4O_7$; 558.7 Da; 279 e^-) and iohexol ($C_{19}H_{26}I_3N_3O_9$; 821.1 Da; 392 e^-) weighed, the amount of Milli-Q water (H_2O; 18 Da; 10 e^-) added, and the measured solvent volumes of the stock solutions according to Eq. S1, and based on the reference values reported in Table S1. The mass of water was calculated from the added volume (assuming 1 mg = 1 µL).

$$\rho_{sol} = \left(\frac{m_{agent}}{MW_{agent}} \cdot N_{e-} + \frac{m_{H2O}}{MW_{H2O}} \cdot 10e^-\right)/V_{final} \qquad \text{(Eq. S1)}$$

Electron densities of buffers and DDM samples at different concentrations were calculated from the reference values in Table S1 by linear extrapolation (using the real concentrations from Table S2) and a water electron density of 0.335 $e^-/Å^3$. The maximum electron densities reached were 0.395 $e^-/Å^3$ for 1,600 mM sucrose, 0.425 $e^-/Å^3$ for 1,470.5 mM Gd-HPDO3A, and 0.455 $e^-/Å^3$ for 1,138 mM iohexol. In order to validate the linear interpolation of electron densities, the mass densities (g/cm^3) of sucrose and iohexol solutions, covering the experimental range of the SAXS experiments, were measured at 20 ºC on an Anton Paar DMA 5000 density meter. To this end, stock solutions of 1.508 M sucrose (512.89 mg + 680 µL Milli-Q, V_{final} = 993 µL) and 1.006 M iohexol (967.27 mg + 710 µL Milli-Q, V_{final} = 1170 µL) were freshly prepared, following the protocol of the original SAXS samples. Dilution series were obtained by addition of appropriate volumes of Milli-Q water. Experimental results are shown in Table S6 and Fig. S7 and demonstrate a perfect linearity of both solutions. Moreover, both densities are in very good agreement with literature (Weast, 1989, Rickwood *et al.*, 1982). We did not

have enough material to prepare a fresh density calibration series of Gd-HPDO3A after the SAXS experiments but the value calculated from the 1 M stock solution (Table S1) is in good agreement with densities reported for ProHance® (https://www.accessdata.fda.gov/drugsatfda_docs/label/2013/020131s026,021489s003lbl.pdf).

SAXS theory, experiments and data reduction

For isotropically oriented and non-interacting (i.e. ideal) particulate systems, the scattered intensity *I*(q) can be written as (Lindner & Zemb, 2002):

$$I(q) = \langle \left| \int (\rho(\vec{r}) - \rho_{sol}) e^{i\vec{q}\cdot\vec{r}} dV \right|^2 \rangle \qquad \text{(Eq. S2)}$$

where $q = \frac{4\pi}{\lambda}\sin\theta$ is the modulus of the scattering vector, λ the X-ray wavelength, and 2θ the scattering angle. The brackets indicate an average over all orientations of the solubilized particles. $\rho(\vec{r})$ and ρ_{sol} are the electron densities of solubilized particles (soft matter systems, biomacromolecules) at a position $\vec{r}$ and the bulk solvent. The integral runs over the whole particle volume *V*.

All SAXS experiments were carried out on the SWING beamline (https://www.synchrotron-soleil.fr/en/beamlines/swing) at the synchrotron SOLEIL (Saint Aubin, France) in flow mode, using an X-ray energy of 12.00 keV and a sample-detector (Aviex CCD) distance of 1.790 m, yielding an exploitable q-range from 0.006 to 0.545 $Å^{-1}$. For each sample a volume of 40 µL circulated at 75 µL/min through a thermalized quartz capillary of 1.5 mm diameter and 10 µm wall thickness, inserted within a vacuum chamber (David & Perez, 2009). Individual 1s time frames were collected at 15 ^{0}C. The 2D scattering patterns were reduced into 1D intensities and selected for averaging using the Foxtrot software (www.xenocs.com). Buffer intensities were subtracted from sample intensities by using the program PRIMUS from the ATSAS program suite (Franke *et al.*, 2017), after a calibration against the measured transmissions.

Final 1D buffer curves were subtracted from the respective 1D sample curves by using the program PRIMUS from the ATSAS suite (Franke *et al.*, 2017), after appropriate calibration against the concentrations of the respective medical contrast agents (Fig. S2). The sample transmissions depended very strongly on the concentrations of the medical contrast agents, thus providing an efficient tool to calibrate their real concentrations C_{real} with respect to the nominal ones C_{nom}. To this end, $\ln\left(I_{before}(C_{nom}) / I_{after}(C_{nom})\right)$ vs. C_{nom} was plotted for each buffer concentration and a linear fit applied (Fig. S3):

$$y = a + bC_{real} \qquad \text{(Eq. S3)}$$

I_{before} and I_{after} are the measured X-ray direct beam intensities by intensity monitoring devices based on light-sensitive diodes before and after (in the beamstop) the samples. Eq. S3 allowed to retrieve the

real concentrations of the medical contrast agents in both the DDM samples and the buffers from the measured transmissions (Table S2). The X-ray transmissions of the sucrose solutions did not display a notable dependence on concentration and no *in situ* calibration could be carried out.

The one-dimensional curves of the DDM samples and the buffers displayed strong inter-particle effects over the whole q-range, originating from the presence of contrast agents (Fig. S2). In order to align the buffer signal with the respective sample and to carry out the buffer subtraction in the most accurate way, we generated calibrated buffers, $I_{buff,calib}(q)$, by a linear combination of the buffers lying above ($I_{buff,above}(q)$) and below ($I_{buff,below}(q)$) the DDM curve of interest (Fig. S2):

$$I_{buff,calib}(q) = \frac{C_{DDM} - C_{buff,below}}{C_{buff,above} - C_{buff,below}} I_{buff,above}(q) + \frac{C_{buff,above} - C_{DDM}}{C_{buff,above} - C_{buff,below}} I_{buff,below}(q) \qquad \text{(Eq. S4)}$$

$C_{buff,above}$ and $C_{buff,below}$ are the concentrations of the buffers above and below the DDM sample with concentration C_{DDM}. Please note that all concentrations in Eq. S4 are the real concentrations, calibrated as described, and not the nominal concentrations.

SAXS data analysis

Basic parameters (forward scattered intensity $I(0)$, radii of gyration $R_{G,}$ maximum dimensions D_{max} and pair distance distribution functions $p(r)$) were extracted from the final 1D scattering curves (Fig. 2, S1, Table S4) by using the Guinier approximation in PRIMUS and the program GNOM from the ATSAS package (Franke *et al.*, 2017). The experimental contrast match points (CMPs, i.e. $I(0) = 0$) of DDM in sucrose, iohexol and Gd-HPDO3A were determined by plotting the square root of the forward scattered intensity, $\sqrt{I(0)}$, vs contrast agent concentration (in mM) and by applying a linear fit (Jacrot, 1976). The intersection of the linear fit function with the abscissa yielded the concentration of the respective contrast agent where the DDM micelles are matched (Fig. S4). By using the experimental values of 1 M reference solutions (Table S1) and assuming a linear dependence of the water and contrast agent masses as a function of concentration, the CMPs of DDM in sucrose, Gd-HPDO3A and iohexol can be expressed as weight/weight fractions: w/w % = mass of contrast agent / total weight. The total weight being the weight of dissolved contrast agent plus water added.

Theoretical electron densities of DDM head- (0.517 $e^-/Å^3$) and tail-groups (0.285 $e^-/Å^3$) were calculated from their chemical compositions ($C_{12}H_{21}O_{11}$ and $C_{12}H_{25}$, respectively) and their molecular volumes (350 and 340 $Å^3$, respectively), according to literature (Breyton *et al.*, 2013).

The aggregation numbers N_{agg} of DDM micelles (Table S5) were determined from the experimental $I(0)$ intensities in absolute units (cm^{-1}), according to the following equation:

$$I(0) = c \cdot MW \cdot (\Delta\rho)^2 / (\rho_m^2 \cdot N_A) \qquad \text{(Eq. S5)}$$

c is the DDM concentration in g/cm^3, MW the molecular weight of a micelle (in Da), $\Delta\rho$ the scattering length density difference between the solvent and DDM (in cm^{-2}), ρ_m the mass density of DDM (in

g/cm^3) and N_A Avogadro's constant. The following values were used for the calculation: c = 0.02 g/cm^3, ρ_m = 1.18 g/cm^3 (from the experimental micelle electron density, determined by the match point, 0.387 e$^-$/Å^3), and N_A = 6.023·10^{23}. The molecular weight of a DDM monomer was calculated from its chemical composition ($C_{24}H_{46}O_{11}$) to be 510.62 Da. $\Delta\rho$ was calculated from the experimental micelle electron density (0.387 e$^-$/Å^3) and the respective solvent electron densities (0.335, 0.395, 0.425 and 0.455 e$^-$/Å^3), by using the equivalence 0.335 e$^-$/Å^3 = 9.40·10^{10} cm^{-2} (Jacrot, 1976).

Ab initio shapes of DDM micelles were calculated by using the multi-phase modeling program MONSA from the ATSAS package (Franke *et al.*, 2017) for a q-range up to 0.25 Å^{-1}, and by assuming a constant density for the head- and tail-groups within the micelles. All electron densities and contrasts used in the MONSA runs were calculated as detailed above and are reported in Table S3. The total micellar head- and tail-group volumes (i.e. MONSA phases) were assumed to be 45,500 and 44,200 Å^3, respectively. MONSA was run in multiple setups (one or multiple contrasts, looseness and contiguity set to 100 or 0%). The models shown (Fig. 2, Fig. S5 and S6) were obtained by using the default values proposed by MONSA based on an initial spherical search volume for a D_{max} from the $p(r)$ analyzes: bead sizes were 2.25 Å, looseness, discontiguity, peripheral and volume fraction penalty weights were 50.00. The initial annealing temperature was 10.00 with an annealing schedule factor of 0.95.

Table S1 Example of preparation protocol of contrast solutions and calculation of solvent electron densities of 1 M reference solutions. V_{final} is the final volume measured by pipetting, C_{final} is the calculated molar concentration of the solution. MW_{agent} and N_{e-} are the molecular mass and the number of electrons per contrast agent molecule, H_2O the volume of Milli-Q water added to the powder mass, m_{agent}, and $ρ_{sol}$ is the solvent electron density calculated by using Eq. S1.

Sample	m_{agent} [mg]	MW_{agent} [Da]	N_{e-}	H_2O [µL]	V_{final} [µL]	C_{final} (M)	$ρ_{sol}$ [$e^-/Å^3$]
Sucrose	399,7	342,3	182	917	1167	1,001	0,372
Gd-HPDO3A	404,2	558,7	279	493	723	1,001	0,396
Iohexol	967,3	821,1	392	710	1170	1,007	0,441

Table S2 Nominal and real (i.e. calibrated) concentrations in mM. Apart from the highest iohexol concentration, errors between nominal and real concentrations are in the range of 2-4%.

Gd-HPDO3A buffer		Gd-HPDO3A DDM		Iohexol buffer		Iohexol DDM	
C_{nom}	C_{real}	C_{nom}	C_{real}	C_{nom}	C_{real}	C_{nom}	C_{real}
250	253	250	247	317	317	317	312
500	522.5	500	517	635	628	635	609
750	738	750	734.5	952	958	952	971
1,250	1,245.5	1,250	1,222	1,270	1,100	1,270	1,138
1,500	1,502	1,500	1,470.5				

Table S3 Contrast for the core (tail) and shell (head) phases of the DDM micelles in MONSA. C_{real} are real (i.e. calibrated) concentrations in mM, contrast is given in 10^{10} m^{-2} (with 0.335 $e^-/Å^3 \approx 9.40 \cdot 10^{10}$ m^{-2} (Jacrot, 1976)).

Sucrose			Gd-HPDO3A			Iohexol		
C_{real}	Shell contrast	Core contrast	C_{real}	Shell contrast	Core contrast	C_{real}	Shell contrast	Core contrast
0	4.66	-1.63	0	4.66	-1.63	0	4.66	-1.63
200	4.43	-1.86	247	4.21	-2.08	312	3.68	-2.61
400	4.21	-2.08	517	3.71	-2.58	971	1.62	-4.67
800	3.76	-2.53	734.5	3.31	-2.98	1,138	1.09	-5.19
1,200	3.31	-2.98	1,222	2.41	-3.88			
1,600	2.86	-3.43	1,470.5	1.95	-4.34			

Table S4 Basic fit parameters of the SAXS contrast series in sucrose, Gd-HPDO3A and iohexol. Radii of gyration R_G were obtained from Guinier fits and *p*(r) distributions, and maximum dimensions D_{max} from *p*(r) distributions by using GNOM from the ATSAS suite (Franke *et al.*, 2017). The errors of the D_{max} are estimated to be about ± 5Å. Please note that some radii of gyration are imaginary, i.e. multiples of the imaginary unit i (with $i^2 = -1$). They result from a negative value of a squared radius of gyration which can occur when some regions of solubilized particles have positive and others have negative contrast (Appolaire *et al.*, 2014, Jacrot, 1976, Stuhrmann, 1974). The respective SAXS curves and Guinier fits (Fig. 2, Fig. S1 and S5) have negative slopes at the origin.

**Sucrose**		
C_{real}	R_G (Å), Guinier/GNOM	D_{max} (Å)
0	30.6±0.0 / 31.1	74
200	32.4±0.0 / 32.2	74
400	34.2±0.1 / 33.7	77
800	40.4±0.2 / 39.7	78
1,200	56.3±13.0 / 60.6	78
1,600	50.8i±4.0i / 42.6i	77

**Gd-HPDO3A**		
C_{real}	R_G (Å), Guinier/GNOM	D_{max} (Å)
0	30.9±0.0 / 31.6	77
247	34.7±0.1 / 33.8	77
517	49.2±1.4 / 43.8	77
734.5	16.9i±120.0i / 28.9i	77
1,222	18.6i±1.1i / 20.3i	77
1,470.5	6.6i±5.6i / 3.8i	77

**Iohexol**		
C_{real}	R_G (Å), Guinier/GNOM	D_{max} (Å)
0	32.4±0.1 / 31.3	76
312	33.3±0.8 / 35.2	74
609	34.0i±12.1i / 38.5i	77
971	26.1±0.5 / 27.2	105
1,138	26.0±0.8 / 28.1	125

Table S5 Apparent DDM micelle aggregation numbers calculated from the *I*(0) intensities according to Eq. S5, based on a common contrast match point of 0.387 $e^-/Å^3$, i.e. a DDM monomer volume of 716 $Å^3$. Δρ is in units of 10^{10} cm^{-2}. The relative variations of 5-6% of the *I*(0) intensities at 0 mM are due to weighing errors of lyophilized DDM powder (1 mg dissolved in 50 µL of Milli-Q water).

**Sample**	***I*(0)**	**Δρ**	N_{agg}
Sucrose (0 mM)	0.370	1.48	139
Sucrose (1,600 mM)	0.0063	-0.20	129
Gd-HPDO3A (0mM)	0.395	1.48	148
Gd-HPDO3A (1,470.5 mM)	0.184	-1.05	137
Iohexol (0 mM)	0.416	1.48	156
Iohexol (1,138 mM)	0.630	-1.89	145

Table S6 Mass densities ρ (g/cm^3) determined for sucrose and iohexol solutions. Each concentration was measured at 20 ºC in triplicate on an Anton Paar DMA 5000 density meter.

$C_{sucrose}$ [mM]	ρ [g/cm^3]	error ρ [g/cm^3]	$C_{iohexol}$ [mM]	ρ [g/cm^3]	error ρ [g/cm^3]
0	0.998136	0.000024	0	0.998136	0.000024
252	1.0315367	0.0002103	252	1.10609	0.00051
503	1.0641563	0.0005713	504	1.2129033	0.0021253
1006	1.128354	0.000243	755	1.3195677	0.0021483
1509	1.192288	0.000787	1007	1.429003	0.000793

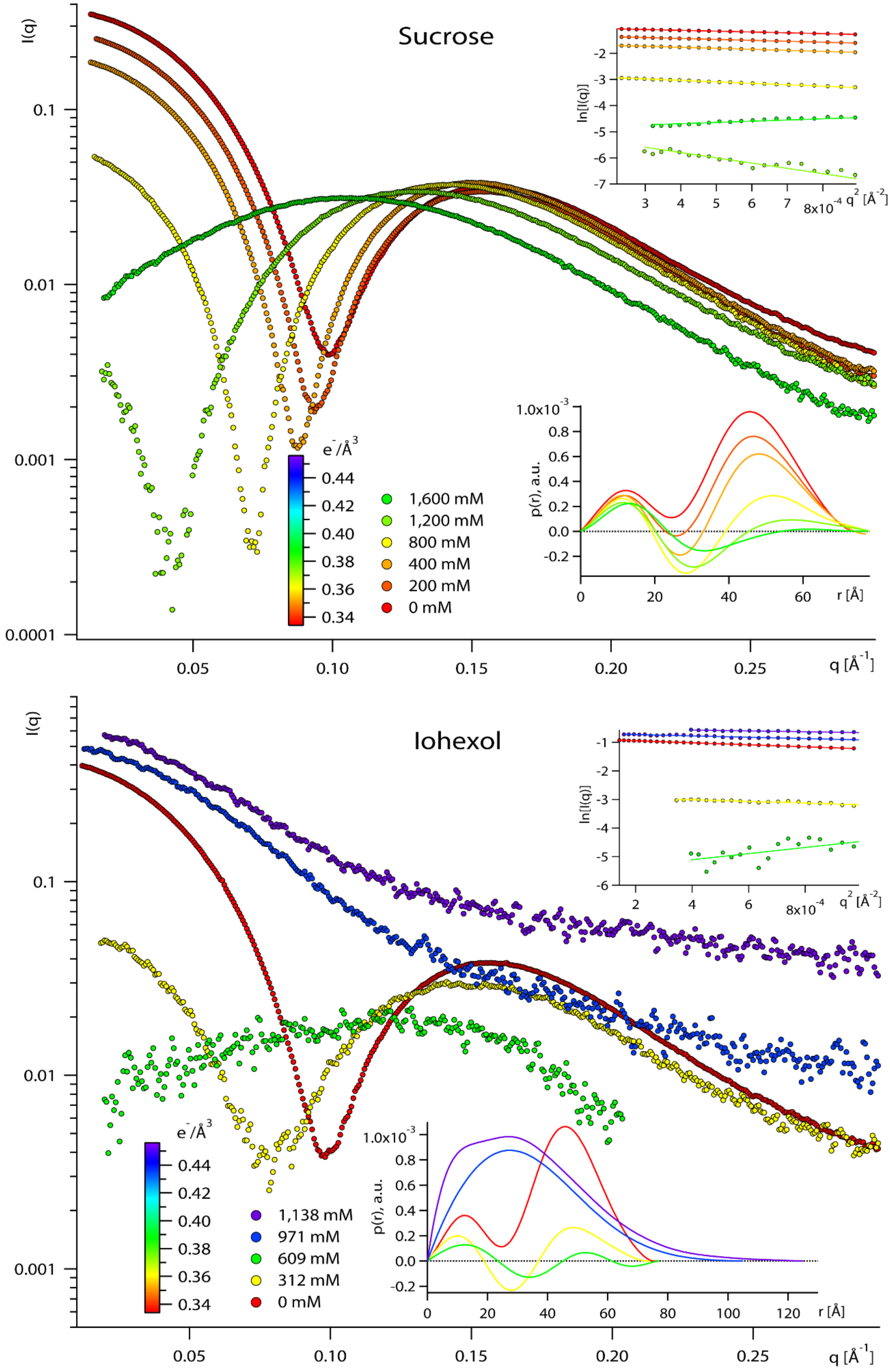

Figure S1 SAXS curves, Guinier fits and pair distance distribution functions p(r) from DDM micelles in sucrose (top) and iohexol (bottom) solutions at various concentrations.

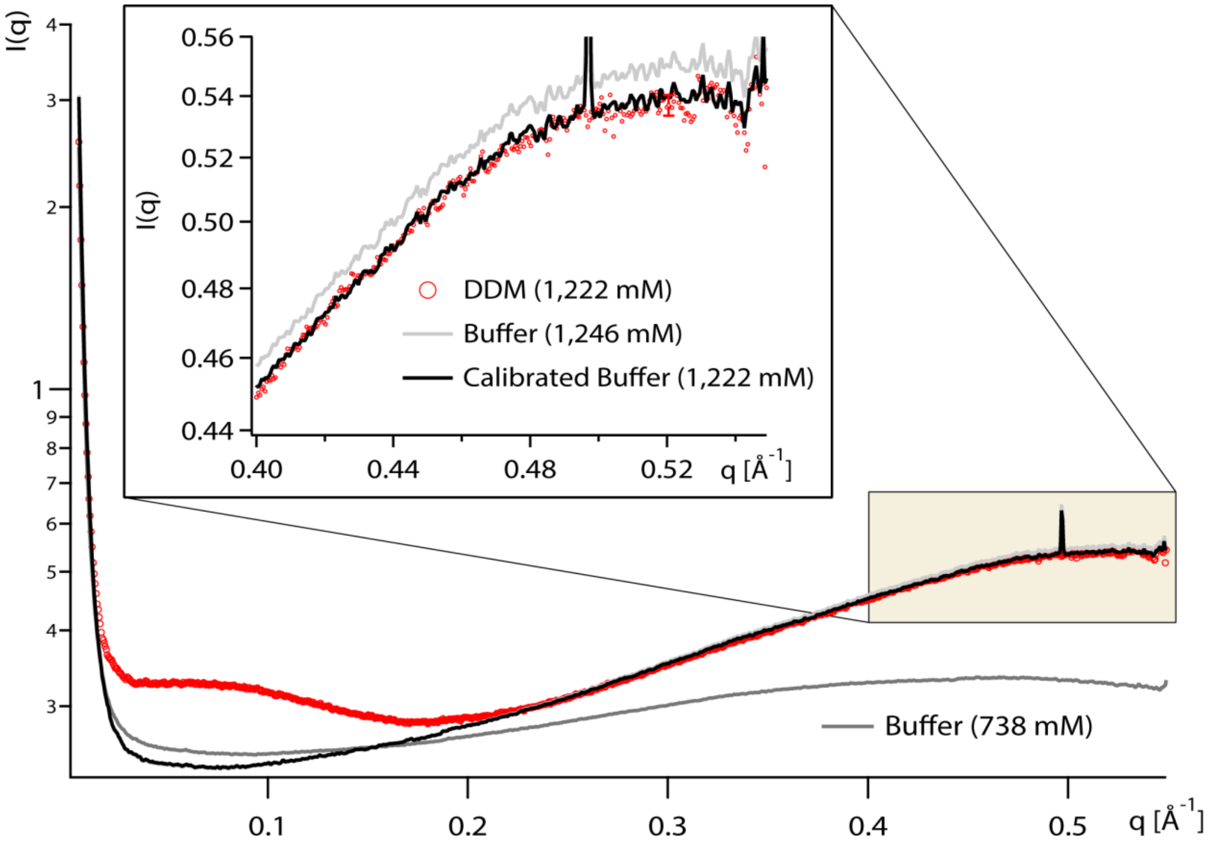

Figure S2 Example of the buffer correction. The plot shows the DDM data in 1,250 mM Gd-HPDO3A (nominal concentration, real concentration 1,222 mM), and the 1,250 and 750 mM Gd-HPDO3A buffers (nominal concentrations, real concentrations 1,246 and 738 mM, respectively). The real concentrations were calculated from the sample transmissions as described (Fig. S3, Eq. S3). The calibrated buffer (black line) was obtained by a linear combination (interpolation) of the 1,250 and 750 mM buffers (Eq. S4). Inset: zoom at the high angular region. A single, representative error bar is shown for q = 0.52 Å^{-1}. While all SAXS data were recorded up to 0.545 Å^{-1}, in most figures and analyses we cut the data at 0.25 Å^{-1}, since beyond that value the buffer and sample intensities were indistinguishable for most datasets (see below).

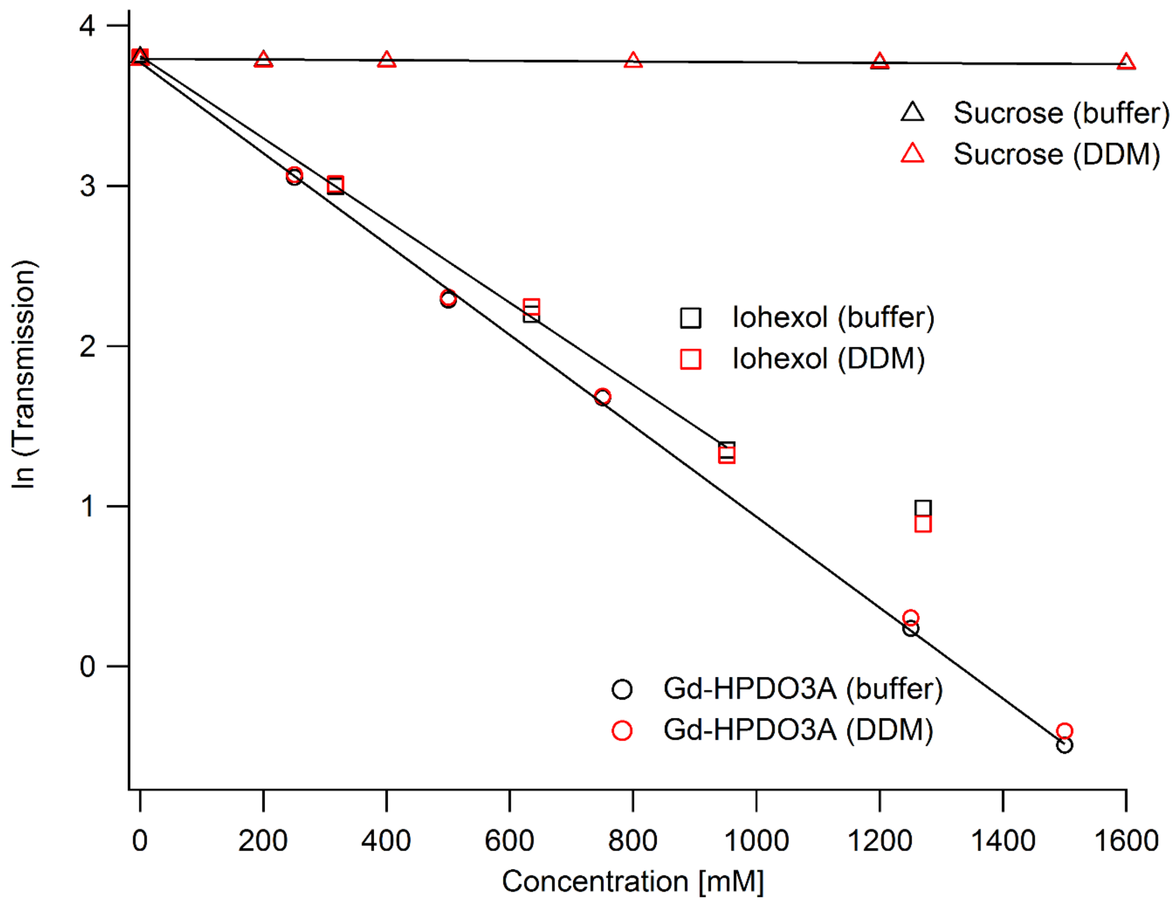

Figure S3 Logarithm of transmissions of sucrose, Gd-HPDO3A and iohexol samples as a function of nominal concentrations. The linear fits were used to calibrate the real concentrations. All data points (concentrations) were used in the case of sucrose and Gd-HPDO3A. In the case of iohexol, the highest concentration was considered to be an outlier and was not used for the fit.

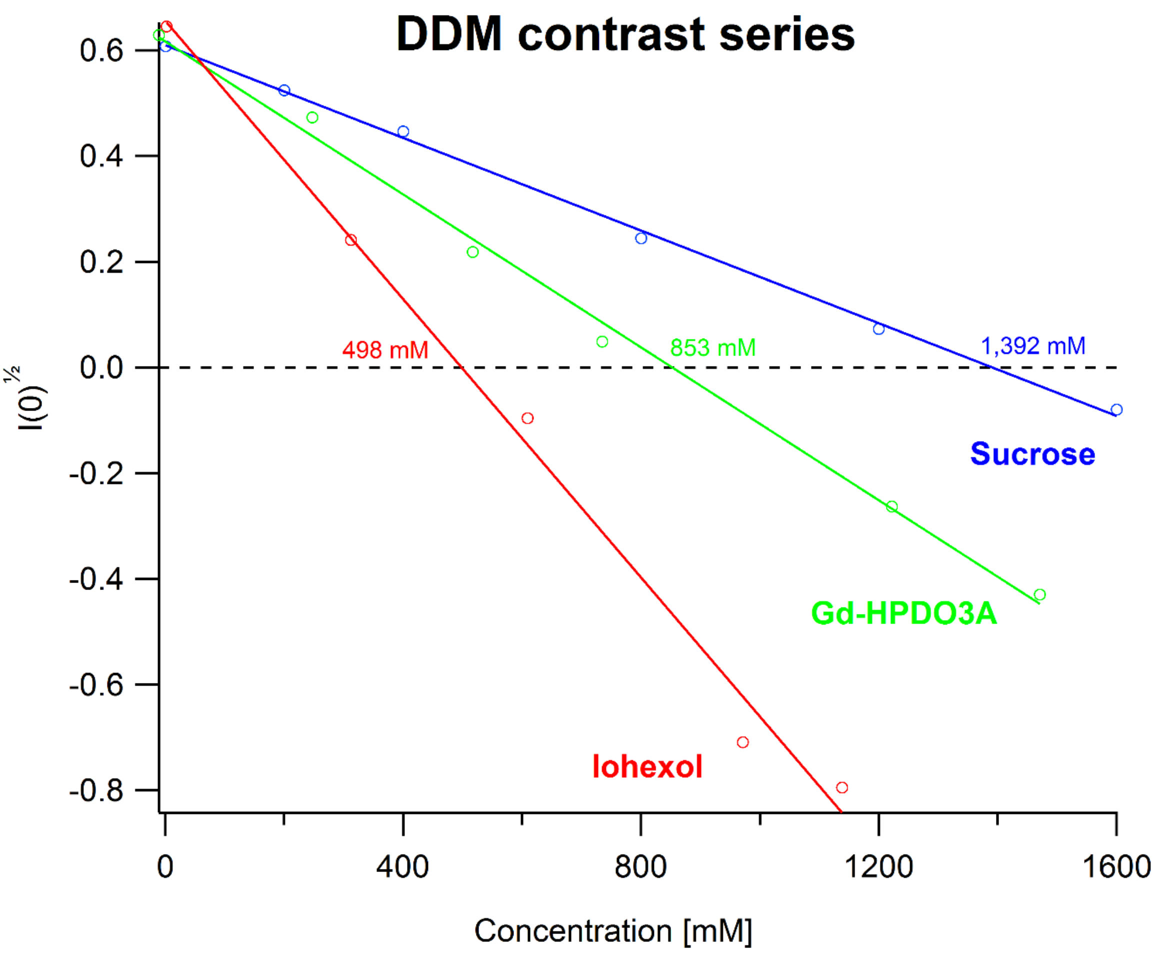

Figure S4 Square roots of the forward scattered intensities $I(0)$ of DDM micelles in sucrose, Gd-HPDO3A and iohexol solutions, as a function of calibrated solute concentration C_{real} (in mM). The values of the intersections of the linear fit functions with the abscissa (broken black line) denote the respective concentrations at the contrast match points (CMPs), i.e. the concentrations where $I(0) = 0$. Based on the relative errors of the contrast concentrations (Table S2) and the relative errors of the DDM concentrations (Table S5), we estimate the relative errors of the CMPs to be about 5 %. The corresponding electron densities were calculated according to equation S1.

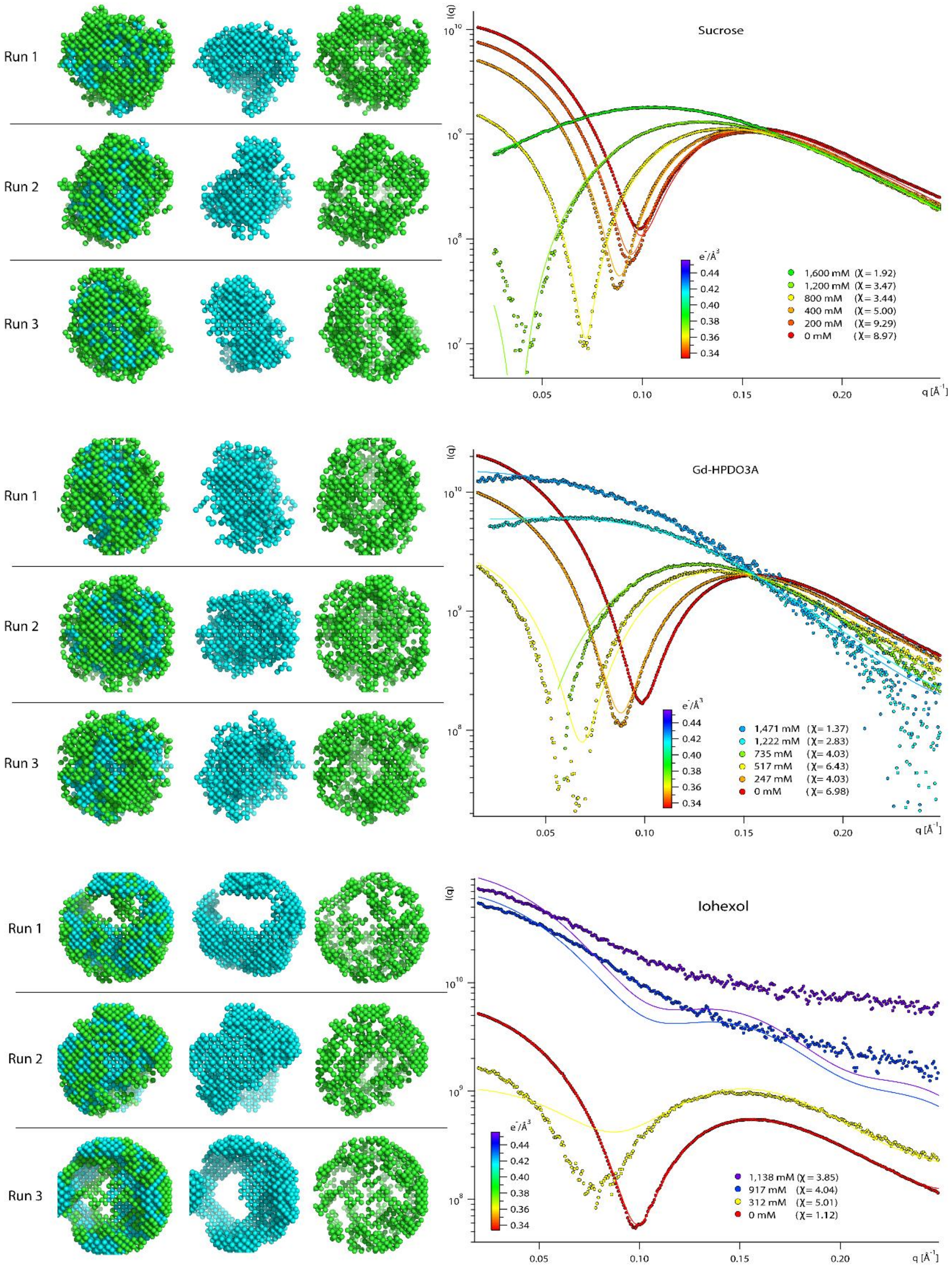

Figure S5 Three distinct MONSA models in default mode for sucrose, Gd-HPDO3A and iohexol, respectively, and by using all SAXS curves. The fit, including χ-values, is from Run 1 in each case. The 609 mM dataset was not used in the case of iohexol since it had a limited q-range and very poor statistics with respect to the four others (see Fig. S1). The largest deviations between MONSA fits and experimental data are observed for contrast curves with the lowest statistical impact: 1,200 mM sucrose, and 517 mM Gd-HPDO3A. (Please note the logarithmic scale of intensities). In the case of iohexol, the MONSA modeling failed.

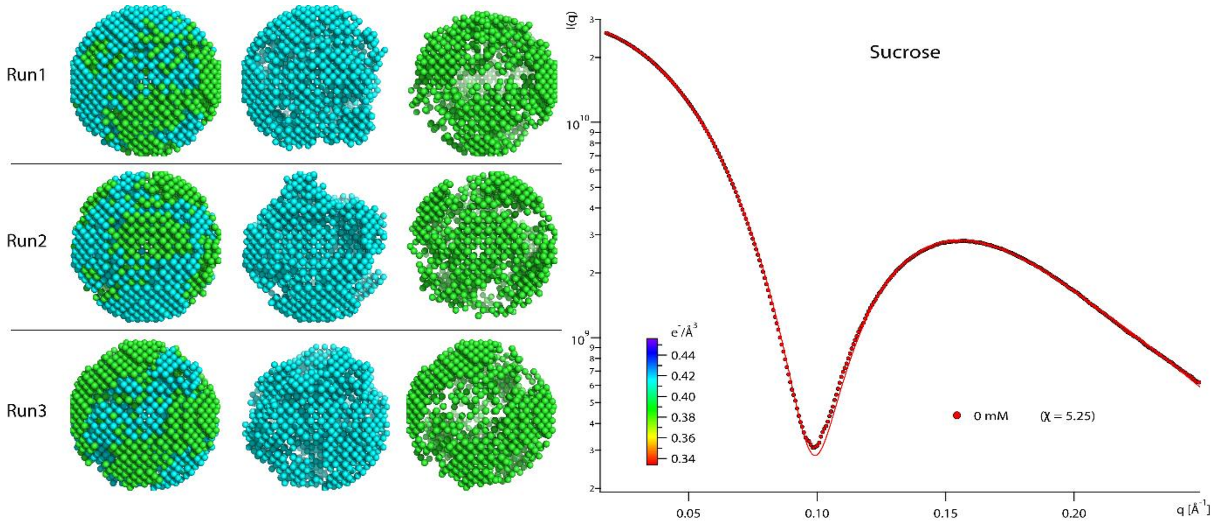

Figure S6 Three distinct MONSA models in default mode for sucrose (0 mM), i.e. by using a single SAXS curve. The fit, including χ-value, is from Run1.

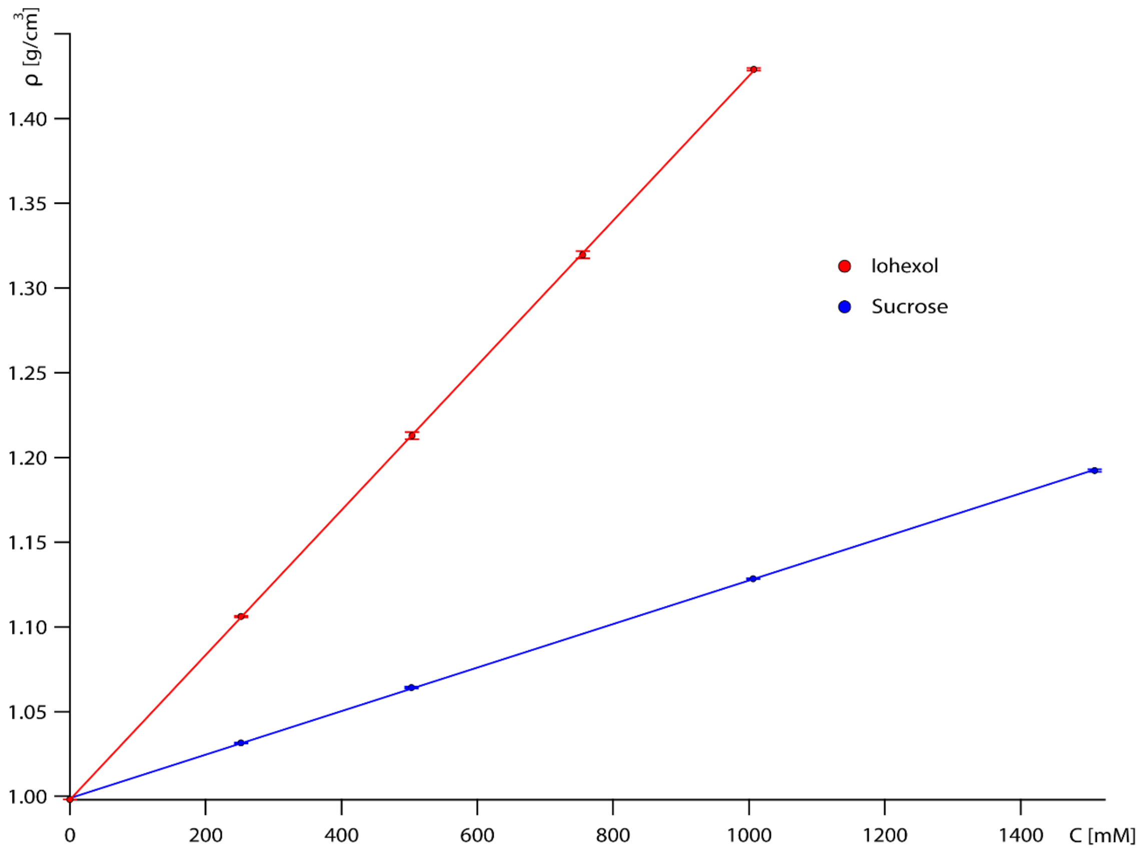

Figure S7 Mass densities ρ (g/cm^3) of sucrose and iohexol solutions in the concentration range covered in the present SAXS study. The values were determined in triplicate at each concentration on an Anton Paar DMA 5000 density meter at 20 °C. The lines represent linear fits.
